# Management of Chronic Pain in Adults Living With Sickle Cell Disease in the Era of the Opioid Epidemic: A Qualitative Study

**DOI:** 10.1001/jamanetworkopen.2019.4410

**Published:** 2019-05-24

**Authors:** Cynthia B. Sinha, Nitya Bakshi, Diana Ross, Lakshmanan Krishnamurti

**Affiliations:** 1 Division of Pediatric Hematology-Oncology-BMT, Emory University, Atlanta, Georgia; 2 Aflac Cancer and Blood Disorders Center, Children’s Healthcare of Atlanta, Atlanta, Georgia; 3 Now with the Department of Pediatric Hematology/Oncology/BMT, Emory University, Aflac Cancer and Blood Disorders Center, Atlanta, Georgia

## Abstract

**Importance:**

The hallmark of sickle cell disease (SCD) is vaso-occlusive pain that may be acute and episodic or may progress to chronic, persistent pain with unpredictable and disabling exacerbations. Patients with SCD rely on opioids almost exclusively for acute and chronic pain management.

**Objective:**

To understand how the current opioid epidemic and subsequent guidelines from the Centers for Disease Control and Prevention are associated with the management of acute and chronic pain for patients with SCD.

**Design, Setting, and Participants:**

Qualitative study using semistructured interview guides. Interviews 1 hour or longer were conducted over the telephone. Participants were adults (aged ≥18 years) diagnosed with SCD who experienced pain on 3 or more days per week recruited from national SCD conferences, symposiums, and 2 sickle cell clinics. Open coding analysis facilitated thematic analysis of interview transcripts. Data collection took place from May 2017 to June 2018.

**Main Outcomes and Measures:**

Participant perspective of any changes to their pain management associated with the 2016 guidelines from the Centers for Disease Control and Prevention.

**Results:**

The 15 adults interviewed had a median (range) age of 32 (21-52) years; 13 (87%) were female; and all were of African American race/ethnicity. Participants reported that recently their opioid prescriptions had become more restrictive, were more closely monitored, and were increasingly difficult to fill in pharmacies. Participants also described increased stigmatization about opioid use and that their medical care was being affected by the physician’s exclusive focus on reducing pain medication use. There was an emerging interest among adult patients in the consideration of the use of alternative therapies, including marijuana, to manage pain.

**Conclusions and Relevance:**

These findings suggest that from the perspective of adults living with SCD, the opioid epidemic may have negatively affected patients’ care by increasing barriers to opioids. Patients reported decreased opioid dosing, increased stigmatization regarding opioid use, physician preoccupation with opioid dosage interfering with comprehensive care, and lack of access to alternative therapies.

## Key Points

**Question:**

Are concerns about the opioid epidemic in the United States and the heightened oversight and restrictions on prescribing opioids associated with changes in the management of acute and chronic pain for patients with sickle cell disease?

**Findings:**

This qualitative study interviewed 15 adults with sickle cell disease. Participants reported that they face increased barriers to the use of opioids for pain management, that their physicians exclusively focus on opioid dosage without establishing a multimodality pain management plan, and that they lack access to nonopioid therapies.

**Meaning:**

Adult patients with sickle cell disease should be included in establishing goals for managing pain and improving functionality using multimodality approaches.

## Teaser

This qualitative study interviews patients with sickle cell disease and identifies their views on how the current US opioid epidemic has affected the management of chronic and acute pain related to their disease.

## Introduction

Sickle cell disease (SCD) is a chronic multisystem disorder associated with vaso-occlusive pain and organ damage, leading to substantial morbidity, impaired health-related quality of life, substantial health care costs, and a high risk of premature mortality.^1^ Acute, episodic vaso-occlusive pain may progress to chronic persistent pain^2^ in more than a third of adults with SCD.^2^ Opioid analgesics are the mainstay for the management of acute and chronic pain in SCD.^2^

In recent years, US federal and state governments have implemented comprehensive measures to address the alarming increase in deaths attributed to the abuse and overdose of prescription opioids.^3,4,5^ In an effort to improve communication between clinicians and patients about the risks and benefits of opioid therapy for chronic pain, improve the safety and effectiveness of pain treatment, and reduce the risks associated with long-term opioid therapy, the Centers for Disease Control and Prevention (CDC) published guidelines in 2016 for prescribing opioid therapy for chronic pain.^3^ Opioid prescriptions, which were declining prior to the publication of the CDC guidelines, have demonstrated a greater decline since its publication.^6^

African American patients are more likely than white patients to be suspected of opioid abuse, subjected to more frequent urine screening, and referred to substance abuse services.^7^ In the United States, SCD predominantly affects African American individuals,^8^ and, thus, patients with SCD may face unique barriers to the management of pain with opioids.

We sought to understand how the concerns about the opioid epidemic and the publication of the CDC guidelines are associated with changes in the management of acute and chronic pain for patients with SCD. Using rigorous qualitative research methods, we sought to obtain the perspective of adult patients with SCD on their experience of pain, their interaction with the health care system, and their perception of how the CDC guidelines affected the management of their acute and chronic pain.

## Methods

We recruited individuals aged 18 years or older with SCD who were attending national conferences and 2 sickle cell clinics and from those who self-referred themselves to this study, which was approved by the Emory University institutional review board. Participants were enrolled following in-person or telephonic verbal consent. One investigator conducted semi-structured qualitative interviews with participants either in person or over the telephone. All participants received a $25 gift card for participation. Results are reported in compliance with the Consolidated Criteria for Reporting Qualitative Research (COREQ) reporting guideline.^9^

The interview guide included prompts concerning diagnosis, childhood, family, school, relationships, work, life, pain, pain management, emergency department (ED) experiences, and the participant’s thoughts concerning the opioid epidemic and how it had influenced their care.

All interviews were transcribed verbatim. We used NVivo software version 11 (QSR International) to perform the line-by-line coding.^10^ Coding concepts were derived from the participant responses and categories developed from these concepts with prevalence determined by content analysis, which quantifies the coding schemes and allows identification of the most prevalent of emerging patterns.^11^ Thematic analysis was then used as a descriptive analytical approach to illustrate the major themes.^11^

To achieve in-depth analysis, we focused on a sample of 15 participants. In qualitative interviewing, a small sample allows for a more authentic account,^12^ that is, understanding how the participants’ point of view addresses our research question. The objective was to understand the constructed view of the participants’ social world rather than generalizing it to the population.^13^ Because rigorous analysis runs concurrently with data collection for concept-gathering studies, smaller sample sizes are advantageous for greater involvement of the investigator.^14^ In qualitative research, robust coding schemes can establish data saturation with concepts ceasing to offer any new perspective on the research question after 6 to 12 interviews.^14^

As themes developed, investigators discussed and substantiated each with examples provided. The lead coder (C.B.S.) developed the initial coding scheme and shared the coding scheme with the second coder (D.R.). The second coder coded 20% of the sample. We then compared the number of occurrences of content and calculated a percentage of agreement. There was 90% agreement between the 2 coders identifying the same content coded.

## Results

We approached 21 potential participants, all of whom initially agreed to participate and provided their contact information. Six individuals who had expressed initial interest were not reachable by telephone or email for consent. Twelve participants were recruited from conferences and 3 from clinics. Once they had consented, no participants dropped out of the study. Two participants whose interviews did not specifically address the opioid epidemic were recontacted for follow-up questions.

We interviewed 15 adults (median [range] age, 32 [21-52] years; 13 [87%] female; all of African American race/ethnicity). Five were employed full time or part time and 10 received Social Security disability benefits. All participants self-reported that their genotype was HbSS, HbSC, and/or HbS-β-thalassemia. Participants were interviewed in person and over the telephone. Across these interview methods, there were no discernible differences in participant narrative. The median (range) length of the participant interviews was 52 minutes (range, 45 minutes to 1.5 hours).

All participants reported pain on 3 or more days per week and had a current prescription for opioids to treat their pain. Regular prescriptions of opioids were started in 12 participants in childhood or adolescence and 3 in adulthood.

Three themes emerged from the analysis. First, participants reported that recently their opioid prescriptions have become more restrictive, more closely monitored, and increasingly difficult to fill in pharmacies. Second, participants believed that their physician’s exclusive focus on reducing pain medication resulted in further stigmatization and decreased attention to patient’s overall medical care. Third, there was an emerging interest among adult patients in the consideration of the use of alternative therapies, including marijuana, to manage pain. In many cases, patients felt forced to pursue nonopioid approaches because of issues with access to opioids.

### Increasing Restrictions and Monitoring of Opioid Use

Participants reported being under increasing pressure to sign a treatment agreement, despite, in some cases, having received opioids to treat pain for several years from the same practice. Overall, 10 participants (67%) currently had a signed agreement with their physicians for opioid prescriptions, 8 of them within the year prior to the interview.

Participants understood that the primary objective of the treatment agreement was to ensure that patients do not sell their opioids or go “doctor shopping.” Participants indicated that while they had conversations with their physician about dependence on opioids and the need for reductions in their opioid prescriptions, they did not have conversations about comprehensive evaluation and management of pain, including the use of alternative therapies.

The reduction in prescriptions resulted in participants rationing their pain medication and being unable to increase their dosage at the onset of a pain crisis or in the presence of a trigger for crisis such as cold weather.

One participant stated, “When something stresses me out maybe they used to do every 2 weeks of getting my medication. Now it’s down to once a month or they [the physician] may say instead of third of the pills [30 pills], we’re only going to give you 15. So, it [the opioid epidemic] has definitely affected me personally and I hate that. Like, I know the wintertime is one of the worse times for me. This winter [state] had one of the worse winters we’ve had.”

Frequently, participants described their treatment history with the phrase “used to” to convey that their regular clinician had recently changed their opioid prescription. Additional barriers included limits to the number of opioid prescriptions permissible by their health insurance company or their pharmacy stocking inadequate supplies of opioids. A participant stated, “There was some law…that had changed earlier this year, they wanted to ask my doctor questions on why I needed so much medicine. I have Medicaid.” Another participant stated, “I went to 10 pharmacies in 1 day to get an oxycodone prescription. They told me that they only order a certain amount.”

Participants believed that opioid prescribing in the ED had also changed. Most participants lacked an established pain protocol for the ED and believed that health care professionals were not well versed in the management of SCD pain. One participant stated, “You have those [ED physicians] that they’re so limited in education that they’re skeptical about following your own protocol. It’s like my doctor wrote that protocol. You’re [ED physician] putting me in harm’s way, but of course they’re not familiar with the dosage you are on, things of that sort, so your safety becomes a prohibiting factor to them. There are specific protocols but they’ll ignore it.”

In the ED, participants also reported having to negotiate for their usual dose of opioids. At times, a shortage of hydromorphone was cited as a reason for reducing the dosage or prescribing another opioid. One participant stated, “Many people that have sickle cell are allergic to morphine and they don’t have Dilaudid so now we have to play Russian roulette and take the morphine. I think it is the worst thing to ever happen, I think it is beyond cruel.”

Participants suspected during the shortage of hydromorphone that the hospital staff was reserving the drug for patients with conditions other than SCD. For example, ED staff advised that they do not have hydromorphone in the ED; however, when the patient with SCD was hospitalized, hydromorphone was available.

### Heightened Stigma and Focus on Pain

Participants observed that the preoccupation with monitoring and limiting prescriptions was preventing their physician from providing comprehensive care, including the management of pain and comprehensive care of their disease. One participant stated, “They just ask me what my pain is. They ask me how my pain is. You okay with just using morphine? Yes. Okay and that’s about it and they take my blood work. That’s all the conversation I have. I’ve never had any real in-depth conversation concerning pain management.”

Participants believed they should have interactive conversations with their physician regarding disease management. Two younger participants reported that they were used to having these conversations with their pediatric hematologist, but they were less likely to occur with their adult hematologist.

The preoccupation with opioid monitoring occurred in the ED as well, with ED physicians, instead of taking patients’ reported pain at face value, preferring to wait for blood work to determine whether the patients were actually “sickling.” Consequently, participants felt they continued to be stigmatized as drug seekers and attributed the stigma to 2 primary factors, lack of knowledge among ED physicians in the management of SCD pain and racial bias. Overall, participants expressed the most dissatisfaction with ED physicians’ lack of SCD-related knowledge. They felt that if the physician and staff knew how to treat SCD complications, then they would not question their complaints of pain and stigmatize them as drug seekers. One participant stated, “In pediatrics they teach us ‘know your numbers, know where you don’t do well, know what medications that you take.’ Like that’s supposed to be a help, but when we go in [the ED] as adults and we do that then it’s like ‘whoa we got a drug seeker here!’ Because I’m only here because I’m seeking drugs…, ‘you got a drug seeker!’”

### Using Alternative Therapies

Participants use nonpharmacological approaches such as hot baths and warm compresses, meditation, reading, praying, music, and art to manage pain and reported interest in other nonpharmacological approaches, such as acupuncture and physical therapy. Frequently, these modalities were cost-prohibitive for individuals with Medicaid. One participant stated, “Yes, I really liked the acupuncture but the crazy thing; my insurance company would not pay for that. Now that’s the part that tripped me out because you guys, they [physicians] don’t want us on opioids like that but yet they’re not willing to pay for alternative medicine, you know. I can’t afford $120 every time I’m in pain [and] want to go get acupuncture.”

Participants also volunteered that marijuana was the most common nonopioid pharmacological approach considered. Six participants reported they had tried or currently use marijuana, in varying frequency, to ease SCD chronic pain. Some participants who used marijuana did so owing to increased restrictions and overall difficulties maintaining opioid therapy. A participant stated, “Yes, the hydrocodone pills. Because once CVS got their first prescription there’s only just that 1 prescription and then I needed a refill or whatever [after taking public transportation to pharmacy]. I had to like go back to my clinic and tell them I need a refill. They have to send it upstairs [and] I had to take it up there myself. Then I have to take the [public transportation] and this paper back to CVS. Then CVS said they’re missing something so I had to go back to my clinic. Honestly, really and truly, I do not use my pain medicine [opioids]…I smoke [marijuana].” For this patient, marijuana offered the least restrictive alternative to manage pain.

Other participants sought marijuana to avoid the adverse effects of opioid therapy. One participant stated, “They had me on 180 milligrams of oxycodone and 30 milligrams of methadone. That was my pain regimen at home every day. I went from a very social person to a very isolated person and I would sleep all the time. I told my family, my pastor, and friends I am really close to [about using marijuana]. I only vape when I am in pain or when I feel it so it is not an everyday thing for me. I won’t say I was able to replace all my medicine except for the muscle relaxer but no more opioids. As of right now, I’m not affected by the opioid epidemic because I took myself out of it.”

Those not using marijuana cited concerns with how to gain access to medical marijuana and concerns with the unknown effects of the drug. A participant stated, “You can get medical marijuana in [home state] but the only problem is that it is going to be a hell of a time finding a doctor who’s going to write you a prescription for it.”

While mostly used for pain, participants also reported that the drug eased their depression and anxiety. Participants also reported that marijuana provides relief without the opioid adverse effects of severe constipation and grogginess and may allow them to reduce or eliminate opioids altogether.

The participants who were interested in learning more about using marijuana were worried about using the drug, especially without physician monitoring. Participants were familiar with the effects of long-term opioid use. Owing to the risk of acute chest syndrome, participants expressed concern about smoking marijuana.

## Discussion

To our knowledge, this is the first investigation to use rigorous qualitative methods to examine the perspective of adult patients with SCD on the impact of the national opioid epidemic and the CDC guidelines on their care and experience in health care settings. In the view of the participants, this has led to decreased access to opioids, increasing stigmatization, and impaired delivery of care, all pushing them to seek alternatives with little guidance or support. Thus, patients experience systemic barriers to managing acute and chronic pain and receiving comprehensive care for their SCD. Participants in this study described how the physician’s lack of understanding about pain from SCD results in lack of sensitivity to patient’s pain, not involving the patient in decision making, and consequently undertreating pain. These findings are concordant with results of survey-based studies examining quality of life for adults living with SCD.^15,16,17,18,19,20^ That most participants who signed an agreement with their physician to maintain opioid therapy only did so in the last year and that they “used to” get more opioid prescriptions in the past suggests a recent change in their care. Thus, our study provides the patient perspective on how concerns about the opioid epidemic may have adversely affected the efforts of patients with SCD to seek medical care and pain management.

The intended audience of the CDC guidelines is primary care physicians prescribing opioids for chronic pain or pain conditions lasting more than 3 months. For circumstances outside the scope of these guidelines, such as receiving care for SCD in an ED, the guidelines recommend that physicians refer to guidelines written specifically for these scenarios. The National Heart, Lung, and Blood Institute guidelines^21^ recommend that patients should receive multiple modalities of care and state that the goal of the opioid component of treatment is to manage pain while maintaining functionality.

The CDC guidelines are intended to be voluntary and are not prescriptive.^3^ They are based on the assessment that there is a lack of evidence demonstrating the long-term benefits of opioids to treat chronic pain, that long-term opioid use is potentially harmful, and that there is some evidence of benefits of nonpharmacologic and nonopioid pharmacologic treatments compared with long-term opioid use. The guidelines recommend that clinicians should consider opioid therapy only if the expected benefits to both pain and function are anticipated to outweigh risks to the patients, and that if opioids are used, they should be combined with nonpharmacologic therapy and nonopioid pharmacologic therapy. Furthermore, they recommend that before starting opioid therapy for chronic pain, clinicians should establish treatment goals with all patients, including realistic goals for pain and function, and only continue opioid therapy if there is clinically meaningful improvement in pain and function that outweighs risks to patient safety. This qualitative study did not provide any evidence that physicians were routinely involving patients in discussions of this type or are combining nonpharmacologic therapy and nonopioid pharmacologic therapy such as nonsteroidal anti-inflammatory drugs, anticonvulsants, and antidepressants. These findings underscore the need for further education of physicians about the intent of the CDC’s recommendations. Clinicians should engage patients in a dialogue to establish goals for pain relief and functionality, jointly identify what works and what does not, and, together, formulate a multimodality approach to pain management. For patients with SCD who receive Medicaid,^22,23^ there is limited access to nonpharmacological therapies because Medicaid may not cover these therapies.^24,25^

Several participants viewed marijuana as a potential means of addressing pain without the adverse effects associated with opioids. Medical marijuana is currently legal in almost all US states, but possession remains a federal offense.^26^ Each state determines the medical conditions allowable and method of consumption for the drug, with only 4 states listing SCD as one of the approved indications. While clinical studies report moderate effectiveness of marijuana in the treatment of various chronic conditions,^27,28,29,30^ including chronic pain, where it may be opioid-sparing,^31^ there is little research on its effectiveness in treating SCD pain. Roberts et al^32^ reported that approximately 40% of participants with SCD report using marijuana. Participants in this study cited lack of access to, the unacceptable adverse effects of, and a desire to be free of opioids as reasons for considering the use of marijuana.

Participants also considered physical therapy and acupuncture as potential treatments for managing pain, but access to these interventions may be limited by lack of coverage by Medicaid.^33^ In 4 states, Medicaid covers acupuncture for back pain and migraine, but no state covers this intervention for SCD.^34,35,36,37^ There are limited data available on the efficacy of acupuncture in the management of pain in SCD.^38^

Individuals self-identifying as white non-Hispanic are more likely to be prescribed and engaged in long-term use of opioids compared with other racial/ethnic groups.^39,40^ Of the 16 000 deaths attributed to opioid overdose in 2013,^41^ only 10 occurred in patients with SCD.^41^ Nonetheless, racial minorities are more likely to be perceived as addicted to opioids and exhibiting drug-seeking behavior in the ED.^42^ Race may influence how health care professionals view and treat patients with SCD^43^ and their attitude toward patients’ accounts of intensity of pain.^44^ Participants in this study believed that their race was an influential factor in negative experiences in the ED and that physicians who were knowledgeable about SCD were less likely to exhibit racial bias toward patients with SCD.^45^

### Limitations

There are several limitations to this study. Female patients were overrepresented in the sample. It is possible that a more representative sample could potentially reveal different themes. Participants were recruited from among the attendees at national sickle cell disease conferences and may have been more activated in regard to disease self-management, had access to more resources, and have been less representative of the general population of patients with SCD. Lacking access to participants’ medical records, the investigative team had no way to independently verify participants’ accounts of experience with opioids.

## Conclusions

This study suggests that from the perspective of adult patients, the opioid epidemic may have negatively affected the care of patients with SCD by increasing barriers to care. Patients reported decreased access to opioids, increased stigmatization regarding opioid use, and physician preoccupation with opioid dosage without attention to multimodality care or alternative therapies. Further research is required to generate a better understanding of the mechanisms underlying the transition from acute episodic to chronic persistent pain in SCD and the management of chronic pain, including the use of nonopioid pharmacological and nonpharmacological approaches.
