# Error in Byline

**DOI:** 10.1001/jamanetworkopen.2019.17637

**Published:** 2019-11-22

In the Original Investigation titled “Management of Chronic Pain in Adults Living With Sickle Cell Disease in the Era of the Opioid Epidemic: A Qualitative Study,”^1^ published May 24, 2019, there was an error in the byline. The academic degrees for Nitya Bakshi were incorrectly listed as “MD, MBBS, MS.” They should have appeared as “MBBS, MS.” This article has been corrected.^1^
